# Nucleic acid-based polymers effective against hepatitis B Virus infection in patients don’t harbor immunostimulatory properties in primary isolated liver cells

**DOI:** 10.1038/srep43838

**Published:** 2017-03-08

**Authors:** Catherine Isabell Real, Melanie Werner, Andreas Paul, Guido Gerken, Joerg Friedrich Schlaak, Andrew Vaillant, Ruth Broering

**Affiliations:** 1 Department of Gastroenterology and Hepatology, University Hospital at the University Duisburg-Essen, Essen, Germany; 2 Department of General-, Visceral- and Transplantation Surgery, University Hospital at the University Duisburg-Essen, Essen, Germany; 3 Evangelisches Klinikum Niederrhein GmbH, Duisburg, Germany; 4 Replicor Inc., Montreal, Quebec, Canada

## Abstract

Nucleic acid polymers (NAPs) block the release of subviral particles from hepatocytes, a mechanism consistent with their antiviral activity against hepatitis B virus (HBV) in patients. Analysis of immunostimulatory properties of NAPs were conducted with several NAP species: REP 2006, the prototypic degenerate NAP [dN]_40_, containing TLR9-stimulatory CpG; REP 2055 a clinically active NAP with a sequence [dAdC]_20_ devoid of CpG content; REP 2139 (also clinically active) and REP 2165 (REP 2055 analogues further rendered immunologically inactive by replacing cytidine with 5-methylcytidine and incorporating 2′-O methylation of riboses). These analyses revealed pro-inflammatory responses in human peripheral blood mononuclear cells with REP 2006 and with REP 2139 and REP 2165 only at high dose but displayed no significant antiviral activity. In primary isolated human hepatocytes and liver sinusoidal endothelial cells no significant inflammatory or antiviral responses were detected for any NAPs. In human Kupffer cells pro-inflammatory activity was observed with REP 2006 and REP 2055, whereas a weak but significant induction of interferon genes was only observed with REP 2006 at the highest concentration. We therefore hypothesize that the antiviral activity of NAPs optimized to treat HBV infection in patients cannot be explained by direct induction of innate antiviral responses.

Nucleic acid polymers (NAPs) act through size dependent and sequence independent amphipathic interactions of single stranded phosphorothioated oligonucleotides. NAPs have been shown to have antiviral activity *in vitro* and *in vivo* in a broad spectrum of viruses where they act as entry inhibitors similar to sulfated glycans by interfering with amphipathic alpha helices conserved in viruses with type 1 fusion glycoproteins (human immunodeficiency virus, herpes simplex virus, cytomegalovirus, lymphocytic choriomeningitis virus) or putatively by interfering with apolipoprotein interactions required for viral fusion with the host cell (hepatitis C virus)^1^. In hepadnaviruses, NAPs have both entry and post-entry antiviral effects^2^ but *in vivo* the entry-inhibitory properties of NAPs do not appear to contribute to their antiviral effects *in vivo*^3^. NAPs have the unique ability to clear the hepatitis B surface antigen (HBsAg) from the circulation, an effect which is derived from the blockage of secretion of HBsAg from infected hepatocytes, potentially by interfering with the release of subviral particles^4^. Both *in vivo* and more recently in human patients with chronic HBV infection, clearance of serum HBsAg leads to unmasking of anti-HBsAg antibodies, clearance of HBV DNA and more importantly the apparent enhancement of the efficacy of immunotherapy to achieve functional control of chronic HBV infection^5^.

Oligonucleotides have the ability to stimulate the innate immune response through a variety of pattern recognition receptors (PRR) including TLR3 (dsRNA), TLR7/8 (ssRNA), TLR9 (CpG DNA), RIG-I (ss and dsRNA), MDA5 (dsRNA) and DAI (dsDNA)^6^,^7^ which function as sensors for viral and bacterial infection. The role of innate immunity in chronic viral hepatitis, primarily focusing on parenchymal and non-parenchymal murine liver cells have been described previously, indicating a diversification of TLR signaling pathways in Kupffer cells (KCs) and liver sinusoidal endothelial cells (LSECs) compared to ‘classical’ antigen-presenting cells, such as myeloid dendritic cells^8^. Assuming, that TLR agonist-induced expression of pro-inflammatory (TNF, IL6, IL1b), antiviral (IFNB1) and anti-inflammatory cytokines (IL10) in murine KCs, LSECs, and hepatocytes is cell-type specific^8^,^9^. It has been demonstrated that activation of the local innate immune system of the liver through TLR ligands has the potential to control HBV replication in a co-culture model *in vitro*^10^,^11^. Although inflammatory responses were observed early *in vivo* experiments with degenerate NAPs (i.e. REP 2006), consistent with activation of the innate response^2^,^12^ the antiviral activities of NAPs containing sequences and naturally occurring nucleotide modifications designed to block recognition by pattern receptors^13^,^14^,^15^,^16^,^17^,^18^,^19^, persist and are not accompanied by pro-inflammatory effects *in vivo* or in human patients^3^,^5^,^12^,^20^. However since many of the antiviral effects of NAP therapy in HBV infection are similar to those observed with immunotherapy, a more rigorous examination of immunostimulatory effects of NAPs optimized for therapeutic use was conducted in primary cultures of human parenchymal and non-parenchymal liver cells and peripheral blood mononuclear cells. Experimental setup and description of NAPs are depicted in (Fig. 1).

## Results

### Lack of cytokine gene upregulation in different liver cells treated with NAPs

Cell quality, identity and NAP uptake by different liver cell types was confirmed by treatment of PHHs, KCs and LSECs with cyanine dye 3 (Cy3)-labelled NAPs (REP 2055 [0.01 μM], REP 2139 [0.05 μM] and REP 2165 [0.05 μM]). Uptake of different NAPs was shown in all liver cell types: a weak fluorescent signal was present in the cytoplasm that was either diffuse or punctate nature and more intense in the perinuclear regions and a stronger fluorescent signal was observed in nuclei (Fig. 2). The extent of NAP uptake cannot be quantified from these data. However the cellular localization of NAP signals seemed to be NAP-dependent and cell type-dependent. These localization patterns may reflect different intracellular sorting of NAPs with different chemical modifications (REP 2006 and REP 2055 are unmodified DNA phosphorothioates while REP 2139 and REP 2165 are 2′Omethyl modified RNA phosphorothioates) as well as cell type-specificities. PHHs, KCs, LSECs and PBMCs were stimulated with DNA-based (REP 2006 and REP 2055) and RNA-based (REP 2139 and REP 2165) NAPs or oligonucleotide-based TLR agonists for 6 h. These TLR agonists are specifically recognized by TLR3 (Poly[I:C]), TLR7/8 (ssRNA [ssRNA40 LyoVec™]) and TLR9 (CpG ssDNA [ODN2216]). RNA was extracted, and gene expression of interleukin 6 (*IL6*), tumor necrosis factor (*TNF*), interleukin 10 (*IL10*), interferon alpha 4 (*IFNA4*), interferon beta 1 (*IFNB1*), interferon gamma (*IFNG*) and interferon lambda 2 (*IFNL2*) was assessed by RT-qPCR. As depicted in (Fig. 3) and (Suppl. Fig. 2), no significant inflammatory or antiviral cytokine gene responses could be detected in NAP-treated PHHs. In contrast, Poly(I:C)-treated PHHs showed significant induction of *IL6, TNF, IFNA4* and *IFNL2*. No significant induction of cytokine genes was observed with the TLR7 agonist (ssRNA) or TLR9 agonist (ODN2216). In NAP- and TLR-treated LSECs comparable cytokine responses were observed similar to those observed in PHH, with the exception that Poly(I:C) did not induce *IFNA4* expression (Fig. 3). In KCs, pro-inflammatory activity was restricted to *TNF* and was only observed after treatment with DNA-based NAPs (REP 2006 and REP 2055). These signals were comparable to those induced by ODN2216 stimulation (Fig. 3). A weak but significant induction of interferon genes (*INFA4* and *IFNL2*) in KCs was only observed with the highest concentration of CpG-containing REP 2006. In PBMCs, treatment with REP 2006, REP 2139 and REP 2165 induced significant but less pronounced pro-inflammatory responses (*IL6* and *TNF*) compared to TLR7/8 or −9 agonists. Treatment with REP 2055 resulted in only a very weak induction of *TNF* expression in PBMCs. The TLR9 agonist significantly induced the expression of interferon genes (*IFNA4 and IFNL2*) in PBMCs, while DNA-based NAPs (REP 2006 and REP 2055) did not. However the RNA-based NAPs REP 2139 and REP 2165 showed a weak but inconsistent elevation in *IFNL2* expression in PBMCs (Fig. 3 Suppl. Fig. 2). In all cell types, the gene expression of *IFNB1* and *IFNG* was not influenced by any NAP treatment, whereas at least one TLR agonist showed the responsiveness of these different cell types to TLR-mediated induction if *IFNB1* and *IFNG* genes (Suppl. Fig. 2). RT-PCR performance and integrity after treatment, as determined by comparison of ACTB mRNA in control and treated cells with the total mean of ACTB expression, were shown not be affected by oligonucleotide exposure in any of the tested cell types (Suppl. Fig. 1A–D).

### Lack of interferon secretion in response to NAP treatment *in vitro*

The gene expression analysis of NAP-treated cells was accompanied by detection of cytokine secretion. Primary human hepatocytes, Kupffer cells and peripheral blood mononuclear cells were stimulated with REP 2006, REP 2055, REP 2139 and REP 2165 or TLR agonists for 24 h. LSECs were not included in this analysis due to limited cell yields and lack of responsiveness on the mRNA expression level (Fig. 3). Supernatants were collected and secretion of IL6, TNF, IL10, IFNA4, IFNB1, IFNG and IFNL2 was quantified by enzyme-linked immunosorbent assay (Fig. 4 and Suppl. Fig. 3). No significant increase of IFN secretion was observed upon NAP treatment in both PHHs and PBMCs. Importantly, stimulation with ODN2216 mediated the secretion of IFNA4 (Fig. 4) in both PBMCs and PHHs. Consistent with the results of gene expression in KCs, secretion of pro-inflammatory TNF was observed after treatment with DNA-based NAPs such as REP 2006 and REP 2055 (Fig. 4). In contrast, a very weak but significant interferon response (IFNL2) was only observed with RNA-based NAPs (REP 2139 and REP 2165) (Fig. 4). During NAP treatment, an accumulation of NAPs in the liver is expected, therefore a high dose (50 μM) treatment was performed in the ELISA setup using the clinically active substances (REP 2055 and REP 2139). High dose treatment in liver cells did not significantly increased cytokine secretion compared to 5 μM dosing (Fig. 4). In PBMCs treatment with low doses of REP 2006 and only high doses of REP 2055 and REP 2139 (50 μM) induced weak but significant TNF secretion that was comparable to that induced by ODN2216. Interestingly, only the high dose of REP 2139 induced IL10 secretion as well (Suppl. Fig. 3). Whereas gene expression of *IL10* could not be induced by any of the tested stimuli, strong secretion of IL10 in liver cells (PHHs, KCs) could be induced with TLR3 and TLR7/8 agonists and in PBMCs with TLR7/8 and TLR9 agonists. NAP-induced IL10 secretion was observed in PHHs (REP 2006, REP 2139 and REP 2195) and PBMCs (REP 2139 and REP 2195), however these signals were weak and not dose dependent. To test whether nucleic acids interfere with ELISA detection of cytokines, spiking experiments were performed for IL6 and TNF detection with one low and one high concentration of the ELISA standard was mixed with different concentrations of NAPs [5 and 50 μM] and TLR ligands (ssRNA40 and CpG ODN2216) prior to detection. No interference with ELISA sensitivity was observed for any tested substance (Suppl. Fig. 1E–H).

An overview of gene expression and cytokine secretion profile of all tested cell types in response to NAP treatment is given in Table 1. Cytokine induction activity is indicated for NAPs which caused a dose dependent induction of cytokine gene expression or secretion that was statistically significant. Exposure with any NAP did not mediate any meaningful induction of cytokine gene induction or secretion in PHHs or LSECs. In KCs only DNA-based NAPs induced cytokine gene induction, and an interferon response was observed only with REP 2006. In PBMCs only pro-inflammatory responses were detectable, and no IFN gene induction and/or secretion was observed (Table 1). An induction of IFNL2 secretion was noted with REP 2165 treatment in KC but this was not considered meaningful (see discussion).

## Discussion

NAPs act primarily via an as yet unresolved post-entry mechanism to block the secretion of HBsAg from infected hepatocytes^1^,^4^. The resulting clearance of serum HBsAg unmasks anti-HBs and further relaxes the direct immunosuppression on adaptive and innate immune responses to HBV infection mediated by HBsAg (reviewed by Kondo *et al*.^21^). The ability to rapidly clear serum HBsAg in most patients is an effect unique to NAPs and is associated with other important antiviral impacts including clearance of HBeAg, appearance of free anti-HBs and anti-HBe antibodies, reductions of serum HBV DNA and clearance of HBsAg, HBcAg, HBV DNA and cccDNA from the liver which have been attributed to immune recovery in the absence of circulating HBsAg^4^,^5^. In cytomegalovirus, hepatitis C virus and hepadnaviral infection, previous *in vivo* studies have established that the antiviral activity of NAPs occur independently of any residual immunostimulatory effects present in NAPs^3^,^12^,^20^ however these studies were performed in mice and ducks. Since many of the secondary antiviral effects of NAP treatment could also be caused by direct oligonucleotide-based immune stimulation via recognition by innate pattern recognition receptors, a more in depth evaluation of the potential immunostimulatory properties of NAPs in humans was conducted using primary human cultures of liver and blood cells.

Cytokine responses (gene induction and secretion) to the non-encpasulated oligonucleotide TLR agonists Poly(I:C) and / or ODN2216 were observed in all cell types indicating that oligonucleotide uptake in the absence of a delivery agent was functional for eliciting an immunostimulatory responses under the tissue culture conditions and experimental paradigms used. Moreover, uptake experiments with Cy3 labeled NAPs demonstrated uptake of NAPs into these cells and transit through the cytoplasm and into the nucleus. Intracellular NAP distribution seemed to dependent on the chemical structure and the cell type. In each case, the NAP localization in a particular cell type provides the best reasonable approximation of intracellular NAP sorting in these cell types *in situ*. Thus, the extent of cytokine response observed with each NAP in each cell type provides a reasonable approximation of the reactivity of NAPs in these cell types *in situ*.

Cynomologus monkeys are an accepted surrogate for modelling the pharmacokinetics and biodistribution of phosphorothioate oligonucleotides in humans^22^,^23^,^24^. Analysis of the plasma PK and tissue distribution of REP 2139 and REP 2165 in cynomolgus monkeys receiving 26 weeks of a weekly 9 mg/kg dose (clinical doses in humans range from 4–8 mg/kg) has been recently been completed (Ingo Roehl, personal communication). This analysis shows very rapid clearance from the blood throughout the dosing, from a C_max_ of 11 μM at the end of infusion to less than 10 nM after 24 hours. Tissue accumulation in the liver exceeds 50 μM only at the 6 month time point for REP 2139 and remains less than 10 μM at the end of 6 months of dosing with REP 2165. These results replicate those observed for liver accumulation in preclinical studies^25^ and are consistent with the known pharmacokinetic and tissue distribution behaviours common to all phosphorothioate oligonucleotides^22^,^26^,^27^. Based on the chemical modifications present in REP 2006 and REP 2055 their intermediate stability relative to REP 2139 and REP 2165^25^ will result in liver accumulation for these NAPs within the limits observed for REP 2139 and REP 2165 but will have the same rapid clearance characteristics from the plasma. In all clinical assessments of REP 2055, REP 2139 and REP 2165 performed to date, substantial HBsAg reductions and or clearance is achieved well within 3 months^5^,^28^,^29^ well before any immunostimulatory or pro-inflammatory activity would be predicted to occur based on pharmacokinetics in non-human primates and the weak pro-inflammatory and immunostimulatory properties of NAPs observed in this study.

REP 2006 is the prototypic degenerate DNA NAP compound which can be recognized by TLR9 and has been shown to be associated with pro-inflammatory side effects in mice and ducks^2^,^12^. As expected, treatment with this NAP did result in significant gene induction of pro-inflammatory and antiviral cytokine genes in KC and secretion of IL10 in PHHs, indicating that NAP uptake could also result in an immunostimulatory response in KCs and PHHs. However, these effects were largely eliminated with REP 2055 and were absent with the modified RNA NAPs REP 2139 and REP 2165. The absence of any meaningful immunostimulatory response in liver cells treated with REP 2055, REP 2139 or REP 2165 is consistent with the absence of CpG motifs in the sequence of these NAPs^30^ and also with the presence of 2′O methyl sugar modification and 5-methylcytidine in REP 2139 and REP 2165, naturally occurring nucleotide modifications known to block pattern receptor recognition of DNA or RNA^13^,^14^,^15^,^16^,^17^,^19^. However, the ELISA data indicated a slight induction of IFNL2 in KC after treatment with REP 2165. This response was still near the limit of detection and only occurred in two out of four KC preparations and could not be seen in the gene expression data set. Thus this response is considered not to be biologically relevant. Discrepancies between cytokine gene expression and secretion (i.e. TNF in KC) may be due to differences in how TLR responses can be manifested. Induction of cytokine gene expression may occur but not translation and secretion. Furthermore, secretion of cytokines may be due to the release of cytokine containing vacuoles, without immediate preceding gene expression. The liver is a site that is involved in systemic tolerance induction. Especially low levels of pathogenic material as well as repetitive exposure to pathogens results in tolerance induction^31^. Primary liver cells isolated from different donors may also differ in their immunogenic/tolerogenic properties, thus also donor specific differences may affect the magnitude of cytokine expression and secretion. These suggestions may explain the imbalance in TNF expression and secretion in stimulated Kupffer cells.

Although the control immunostimulatory compounds are specific for TLRs that recognize pathogen associated molecular pattern (PAMP) in endosomal vesicles, they induce similar cytokine responses as cytosolic PAMP receptors such as RIG-I, DAI and MDA-5 which include a type I interferon response^7^,^32^,^33^. REP 2006 was able to stimulate a type I interferon gene induction (although no enhanced secretion was observed) which could be derived from recognition by both endosomal and cytosolic PAMP receptors. The lack of any observable type 1 interferon response (INFA4 and INFB1) with REP 2055, REP 2139 or REP 2165 in any cell type suggests that the cytoplasmic transit of these NAPs does not stimulate cytoplasmic or endosomal PAMP receptors. Antiviral activity of TLR-stimulated parenchymal and non-parenchymal human liver cells seems to be restricted to TLR3 *in vitro*^34^,^35^. Cytosolic pathogen recognition receptors like RIG-I, STING and cGAS can be activated in human hepatocytes as well, leading to antiviral signaling^36^,^37^. However, NAP-treated PHH, KC and LSEC did not induce the expression and secretion of interferons, except KC treated with immunostimulatory REP 2006. Here slight but significant induction of IFNA4 and IFNL2 gene expression was observed that did not result in interferon secretion. The antiviral effects of NAPs, namely the reduction of serum HBV surface antigen in HBV-infected patients are very similar for REP 2055, REP 2139 and REP 2165^5^,^28^,^29^ despite the absence (REP 2055) or presence (REP 2139 and REP 2165) of 2′OMe and 5MeC modifications. It should be noted that although an increase in inflammatory cytokine gene expression in PBMCs is significant for all NAPs, cytokine secretion was only weakly stimulated for REP 2055 and REP 2139 (and not with REP 2165) and was only observed with NAP concentrations about 5-fold greater than their C_max_ in cymologus monkeys, which is short lived. This suggests that paracrine effects in the liver from stimulation of PBMCs by NAPs are very unlikely. Indeed, elevation of serum cytokines are observed in all patients receiving REP 2139 in the REP 102 protocol^38^ but these elevations are not correlated with a reduction in serum HBsAg.

In PBMCs, all NAPs, regardless of sequence composition or modification, elicited significant responses in both pro-inflammatory (TNF and IL6) and anti-inflammatory (IL10) cytokines (gene induction and / or cytokine secretion) with no detectable interferon responses, suggesting that complete suppression of cytokine responses, even with the optimized NAPs REP 2139 and REP 2165 is difficult to achieve for oligonucleotides. However like other phosphorothioated oligonucleotides, NAPs are rapidly cleared from the blood after administration and mainly accumulate in the kidney, liver, spleen and lung^22^,^26^. Moreover, cytokine responses in PBMCs were only observed with 50 μM of REP 2055 or REP 2139, a concentration that is substantially higher than the highest concentrations of NAPs present in the blood at therapeutic doses with these NAPs, as predicted by pharmacokinetic studies in non-human primates^22^.

These studies are in agreement with previously published data indicating that the antiviral activity of NAPs is not derived from a cytokine response directly induced by PAMP recognition of NAPs^2^,^4^ and strongly suggest that the antiviral effects of NAPs in human patients occur independently of cytokine induction.

## Materials and Methods

### Materials

**Figure 1:**
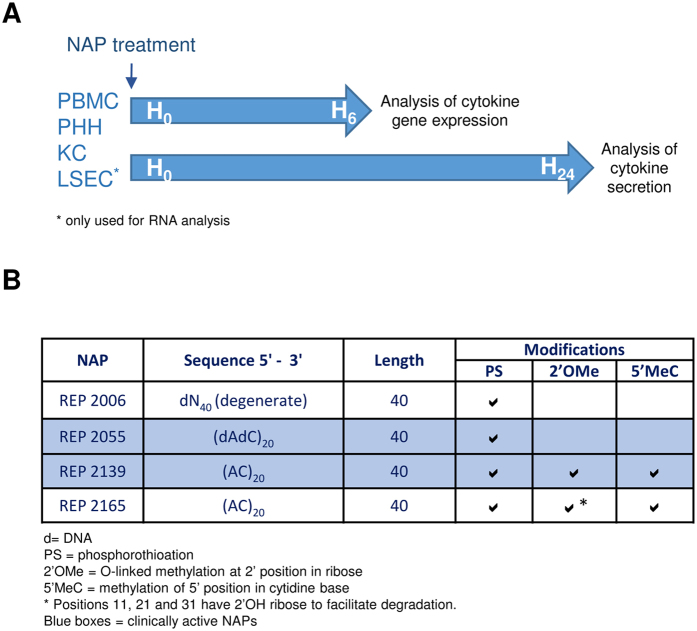
Schematic experimental procedure and NAP description. Primary human hepatocytes (PHH), Kupffer cells (KC), liver sinusoidal endothelial cells (LSEC) and peripheral blood mononuclear cells (PBMC) were stimulated with NAPs for 6 h to analyze cytokine gene expression by qRT-PCR and for 24 h to analyze cytokine secretion by ELISA (**A**). Overview of nucleic acid polymers (NAPs) used in this study (**B**).

**Figure 2:**
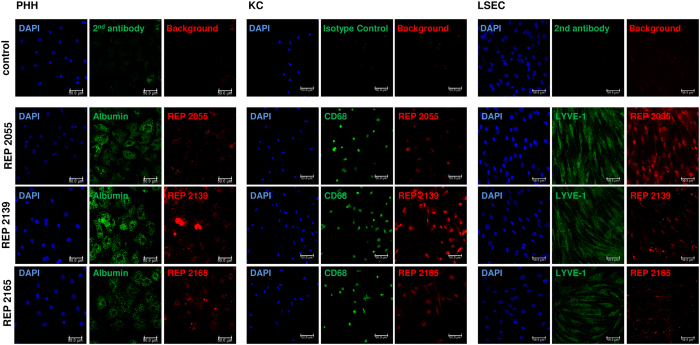
Nucleic acid polymers (NAPs) were efficiently taken up by different liver cell types. The identity of primary human hepatocytes (PHH) (**A**), Kupffer cells (KC) (**B**) and liver sinusoidal endothelial cells (LSEC) (**C**) was assessed by immunofluorescent staining of cell type-specific markers albumin (**A**), CD68 (**B**) and LYVE-1 (**C**) (green), respectively. Nuclei were counterstained with DAPI (blue). Uptake of NAPs was visualized using Cy3-labeld (red) REP 2055 [0.01 μM], REP 2139 [0.05 μM] and REP 2165 [0.05 μM]. Immunofluorescence staining was detected with a laser scanning microscope (LSM; Axiovert 100 M; Zeiss, Jena, Germany) at 20 × magnification. Image analysis was performed with LSM Image Browser (Zeiss). Scale bar 50 μm.

**Figure 3:**
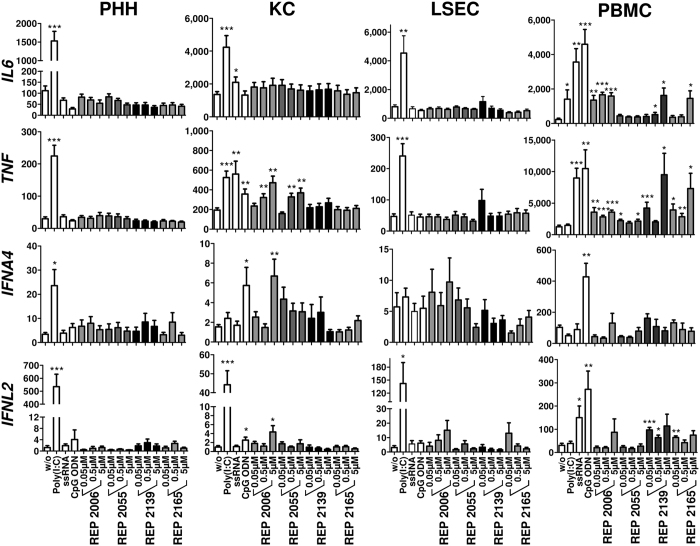
Cell type-specific expression of innate immune genes in response to NAP treatment *in vitro*. Primary human hepatocytes (PHH, n = 3–5), Kupffer cells (KC, n = 3–5), liver sinusoidal endothelial cells (LSEC, n = 3–6) and peripheral blood mononuclear cells (PBMC, n = 3–6) were stimulated with DNA-based (REP 2006 and REP 2055) and RNA-based (REP 2139 and REP 2165) NAPs or immunostimulatory controls (TLR3 agonist Poly(I:C); TLR7/8 agonist ssRNA40 [ssRNA] and TLR9 agonist CpG ODN2216 [CpG ODN]) for 6 h. RNA was extracted, and gene expression of interleukin 6 (IL6), tumor necrosis factor (TNF), interferon alpha 4 (IFNA4) and interferon lambda 2 (IFNL2) was assessed by quantitative reverse transcription polymerase chain reaction (qRT-PCR). Values represented mean ± SEM (normalized to 100,000 copies of beta actin (ACTB) mRNA). Group size n = 3–6 cell preparations. Statistically significant changes compared to untreated controls are reported for p < 0.05 (*), p < 0.01 (**), p < 0.001 (***); w/o, without treatment.

**Figure 4:**
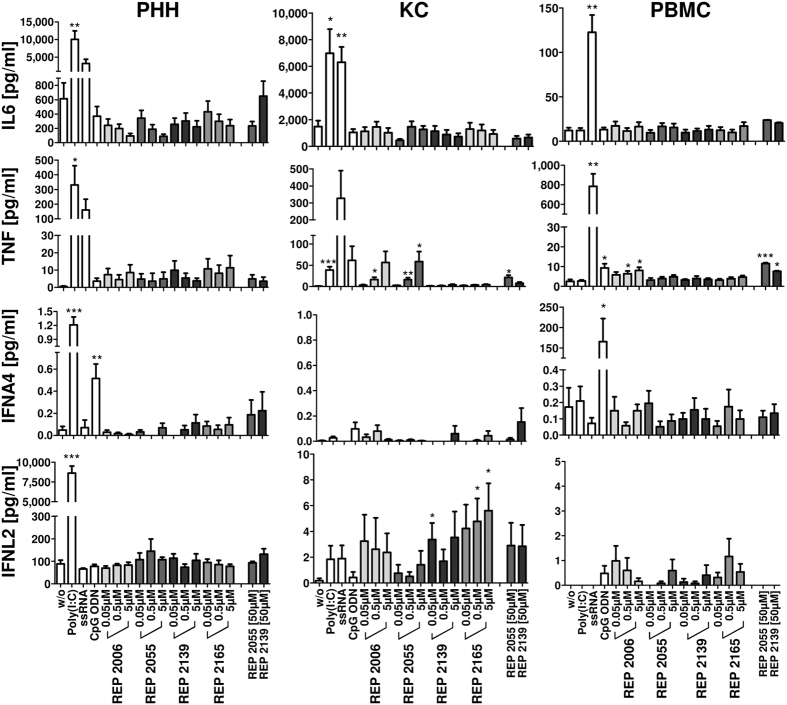
Cell type-specific cytokine secretion in response to NAP treatment *in vitro*. Primary human hepatocytes (PHH, n = 3–5), Kupffer cells (KC, n = 3–6) and peripheral blood mononuclear cells (PBMC, n = 3) were stimulated with DNA-based (REP 2006 and REP 2055) and RNA-based (REP 2139 and REP 2165) NAPs or immunostimulatory controls (TLR3 agonist Poly(I:C); TLR7/8 agonist ssRNA40 [ssRNA] and TLR9 agonist CpG ODN2216 [CpG ODN]) for 24 h. Supernatants were collected and secretion of interleukin 6 (IL6), tumor necrosis factor (TNF), interferon alpha 4 (IFNA4) and interferon lambda 2 (IFNL2) was quantified by enzyme-linked immunosorbent assay (ELISA). Values represented mean ± SEM. Group size n = 3–6 cell preparations. Statistically significant changes compared to untreated controls are reported for p < 0.05 (*), p < 0.01 (**), p < 0.001 (***); w/o, without treatment.

The nucleic acid polymers (NAPs) that were used for the studies were provided by Replicor Inc. (Montreal, Quebec, Canada) as purified sodium salts that were synthesized and purified under cGMP and are described in (Fig. 1). Chemical structures of these NAPs are presented in detail, elsewhere^1^. Briefly, REP 2006 is the prototypic DNA-based 40mer NAP [dN]_40_, that contains residual pro-inflammatory active CpG motifs and has been shown to have significant pro-inflammatory side effects *in vivo*^3^,^12^. REP 2055 is also a DNA-based, 40mer NAP. Its sequence [dAdC]_20_ is devoid of CpG content. This compound is active *in vivo* against DHBV and HCV and clinically active against HBV infection^5^ and displays minimal pro-inflammatory effects^3^,^20^. RNA-based analogues REP 2139 and REP 2165 are variants of REP 2055 further rendered immunologically inactive by replacing cytidine with 5-methylcytidine and the addition of 2′O methylation of the ribose in each nucleotide. REP 2139 has also been shown to have similar antiviral effects as REP 2055 against chronic HBV infection^5^. Oligonucleotide-based ligands specifically recognized by TLR3 (Poly[I:C], HMW), TLR 7/8 (single stranded RNA: ssRNA40\LyoVec™) and TLR9 (CpG motif-containing ssDNA ODN2216) were obtained from Invivogen (Toulouse, France). Final concentrations of TLR agonists used in treatment controls was 0.05 nM (PolyI;C), 5 μM (ODN2216) and 1.4 μM ([ssRNA40] in ssRNA40\LyoVec™).

### Isolation of human peripheral blood mononuclear cells (PBMCs)

Human peripheral blood mononuclear cells (PBMCs) were isolated from healthy volunteers (n = 5) after informed consent and in accordance with the ethical guidelines of the 1975 Declaration of Helsinki, as described elsewhere^39^. Cells were seeded in 12-well plates and were taken into experiment one day after preparation.

### Isolation and culture of human hepatocytes and non-parenchymal liver cells NPCs

Primary human hepatocytes (PHH) and non-parenchymal liver cells (KCs, LSECs) were prepared from non-tumor liver tissue obtained from fresh tumor resections, as described in detail by *Werner et al*.^40^. Purity and functionality of the diverse liver cell types were frequently controlled by methods described in Werner *et al*.^40^. All patients provided written documentation of informed consent. The study conforms to the ethical guidelines of the 1975 Declaration of Helsinki and was approved by the Institutional Review Board (Ethics Committee, statement ID 12-5232-BO) of the medical faculty at the University Duisburg-Essen. Hepatocytes were seeded into collagen-I-coated culture plates using DMEM Ham’s F12 (PAA, Pasching, Austria) supplemented with 10% FCS (PAA), 2mM L-glutamine (PAA) and 100U/ml penicillin, 0.1 mg/ml streptomycin (PAA). PHH were cultured for 24 h, the medium was changed and cells were were taken into experiment. Kupffer cells were seeded into uncoated 24-well plates using DMEM high glucose supplemented with 10% FCS, 100 U/ml penicillin, 0.1 mg/ml streptomycin, and 2mM L-glutamine and were taken into experiment 7 days after preparation. Liver sinusoidal endothelial cells were seeded into collagen-I-coated 5 cm dishes, cultured in in Endothelial Growth Medium 2 (PromoCell, Heidelberg, Germany) containing provided supplements, 100U/ml penicillin, and 0.1 mg/ml for three days, trypsinized and seeded into 24-well plates to proceed with the experiment after cells reached confluence.

### RNA isolation and quantitative RT-PCR

All types of cells (PHH (n = 3–5), KC (n = 3–5), LSEC (n = 3–5) and PBMCs (n = 3–5)) were treated with the different concentrations (0.05, 0.5 and 5 μM) of NAPs and TLR ligands Poly(I:C) [25 μg/ml], ssRNA40, [10 μg/ml] and ODN2216 [2 μM]. Cells were harvest 6 h after treatment (scheme shown in Fig. 1A). Total RNA was extracted and purified with QIAzol™ Lysis Reagent (Qiagen, Hilden, Germany) and the RNeasy Mini Kit (Qiagen) according to the manufacturer’s instructions and gene expression analysis was performed considering MIQE guidelines^41^. One-step quantitative reverse transcription polymerase chain reaction (qRT-PCR) was performed using QuantiFast SYBR Green RT-PCR Kit (Qiagen) using 0.1 to 0.3 μg of total RNA. Expression of all genes (*IFNA4, IFNB1, IFNG, IFNL2, TNF, IL6* and *IL10*) was detected by commercially available primer sets (QuantiTec Primer Assay, Qiagen). The calculated copy numbers were normalized to the human housekeeping gene beta actin (ACTB) which was detected with the sense primer 5′-TCCCTGGAGAAGAGCTACGA-3′ and the antisense primer 5′-AGCACTGTGTTGGCGTACAG-3′. Data are given in mean ± standard error of the mean (SEM). Untreated controls indicate basal expression levels.

### Enzyme-linked immunosorbent *assay* (ELISA)

Cell culture supernatants from human PHHs (n = 3–5), KCs (n = 3–5) and PBMCs (n = 3–5) were collected 24 h after treatment with the different concentrations (0.05, 0.5, 5 and 50 μM) of NAPs and TLR ligands Poly(I:C) [25 μg/ml], ssRNA40, [10 μg/ml] and ODN2216 [2 μM] (scheme shown in Fig. 1A). Cytokine secretion of IFNA4, IFNB1, IFNG, IFNL2, TNF, IL6 and IL10 were determined by ELISA according to the manufacturer’s instructions (IFNA4, IFNB1, IFNG, TNF, IL6, IL10 [R&D Systems, Wiesbaden, Germany]; IFNL2 [IL28A, RayBiotech Inc, Norcross, GA, USA]). Untreated controls represent basal cytokine secretion.

### Microscopic analyses of immunofluorescent staining

The implementation of immunofluorescent staining was described in detail by Werner *et al*.^40^. The identity of the cell types (PHH, KC and LSEC) was confirmed by immunofluorescent staining of the cell type-specific markers: albumin for PHHs (primary monoclonal mouse anti-albumin antibody [clone 188835, R&D Systems, Wiesbaden, Germany]), CD68 for KCs (monoclonal mouse CD68 antibody, FITC-conjugated, clone Y1/82A, Miltenyi Biotec, Bergisch Gladbach, Germany) and LYVE-1 for LSECs (polyclonal rabbit anti-LYVE-I antibody, Santa Cruz, Heidelberg, Germany). Monoclonal mouse IgG2b-FITC isotype control was also used (clone IS6-11E5.11, Sigma-Aldrich-Inc., Saint-Louis, MO, USA). Donkey anti-rabbit IgG (DyLight 488) and donkey anti-mouse IgG (DyLight 488) were used as secondary antibodies (Thermo fisher scientific, Schwerte, Germany). Nuclei were counterstained with 4′,6-diamidino-2-phenylindole (DAPI; Invitrogen, Carlsbad, CA, USA). Uptake of NAPs was visualized using Cy3-labeld REP 2055 [0.01 μM], REP 2139 [0.05 μM] and REP 2165 [0.05 μM]. Fluorescent images were acquired using Axiovent 100 M microscope (Carl Zeiss, Jena, Germany).

### Statistical analysis

Data are expressed as mean ± SEM (standard error of mean). Statistically significant differences between two groups were determined with the Wilcoxon test. Statistical significance was set at the level of p < 0.05.

## Additional Information

**How to cite this article:** Real, C. I. *et al*. Nucleic acid-based polymers effective against hepatitis B Virus infection in patients don’t harbor immunostimulatory properties in primary isolated liver cells. *Sci. Rep.* **7**, 43838; doi: 10.1038/srep43838 (2017).

**Publisher's note:** Springer Nature remains neutral with regard to jurisdictional claims in published maps and institutional affiliations.

## Supplementary Material

Supplemental Figures

## Figures and Tables

**Table 1:** Summary of significant and dose-dependent cytokine responses mediated by NAP treatment.

Cell Type	NAP [5 μM]	Cytokine gene induction							Cytokine secretion						
		*TNF*	*IL6*	*IL10*	*INFA4*	*INFB1*	*INFG*	*INFL2*	TNF	IL6	IL10	INFA4	INFB1	INFG	INFL2
**PHH**	REP 2006										≤4				
	REP 2055														
	REP 2139														
	REP 2165														
**KC**	REP 2006	≤4			≤6			≤6							
	REP 2055	≤2							≤8						
	REP 2139														
	REP 2165														≥8*
**LSEC**	REP 2006								n.e.	n.e.	n.e.	n.e.	n.e.	n.e.	n.e.
	REP 2055								n.e.	n.e.	n.e.	n.e.	n.e.	n.e.	n.e.
	REP 2139								n.e.	n.e.	n.e.	n.e.	n.e.	n.e.	n.e.
	REP 2165								n.e.	n.e.	n.e.	n.e.	n.e.	n.e.	n.e.
**PMBC**	REP 2006	≤4	≤8						≤4						
	REP 2055	≤2							≤6#						
	REP 2139	≤8	≤8						≤4#		≤4#				
	REP 2165	≤6	≤8												

Gene expression and protein secretion of cytokines was analyzed in primary human hepatocytes (PHH), Kupffer cells (KC), liver sinusoidal endothelial cells (LSEC) and peripheral blood mononuclear cells (PBMC) exposure to indicated NAPs [5 μM]. Numbers indicate statistically significant and dose-related fold induction in cytokine gene expression (left, 6 h exposure) or cytokine secretion (right, 24 h exposure) relative to untreated cells. #, significant increase was only determined for 50 μM high dose treatment; *, not considered biologically relevant (see discussion); n.e., not evaluated.

## References

[b1] Vaillant A. Nucleic acid polymers: Broad spectrum antiviral activity, antiviral mechanisms and optimization for the treatment of hepatitis B and hepatitis D infection. Antiviral Research 133, 32–40 (2016). 27400989 10.1016/j.antiviral.2016.07.004

[b2] Noordeen F., Vaillant A. & Jilbert A. R. Nucleic acid polymers inhibit duck hepatitis B virus infection *in vitro*. Antimicrob Agents Chemother 57, 5291–5298 (2013). 23939902 10.1128/AAC.01003-13 PMC3811297

[b3] Noordeen F., Vaillant A. & Jilbert A. R. Nucleic acid polymers prevent the establishment of duck hepatitis B virus infection *in vivo*. Antimicrob Agents Chemother 57 (2013). 10.1128/AAC.01005-13 PMC3811296 23939904

[b4] Noordeen F. . Therapeutic Antiviral Effect of the Nucleic Acid Polymer REP 2055 against Persistent Duck Hepatitis B Virus Infection. PLoS One 10, e0140909 (2015). 26560490 10.1371/journal.pone.0140909 PMC4641618

[b5] Al-Mahtab M., Bazinet M. & Vaillant A. Safety and Efficacy of Nucleic Acid Polymers in Monotherapy and Combined with Immunotherapy in Treatment-Naive Bangladeshi Patients with HBeAg+ Chronic Hepatitis B Infection. PLoS One 11, e0156667 (2016). 27257978 10.1371/journal.pone.0156667 PMC4892580

[b6] Kawai T. & Akira S. The roles of TLRs, RLRs and NLRs in pathogen recognition. Int Immunol 21, 317–337 (2009). 19246554 10.1093/intimm/dxp017 PMC2721684

[b7] Takaoka A. . DAI (DLM-1/ZBP1) is a cytosolic DNA sensor and an activator of innate immune response. Nature 448, 501–505 (2007). 17618271 10.1038/nature06013

[b8] Wu J. . Toll-like receptor-induced innate immune responses in non-parenchymal liver cells are cell type-specific. Immunology 129, 363–374, doi: IMM3179[pii];10.1111/j.1365-2567.2009.03179.x [doi] (2010). 19922426 10.1111/j.1365-2567.2009.03179.x PMC2826681

[b9] Broering R. . Corticosteroids shift the Toll-like receptor response pattern of primary-isolated murine liver cells from an inflammatory to an anti-inflammatory state. Int. Immunol 23, 537–544 (2011). 21750146 10.1093/intimm/dxr048

[b10] Wu J. . Toll-like receptor-mediated control of HBV replication by nonparenchymal liver cells in mice. Hepatology 46, 1769–1778 (2007). 17929296 10.1002/hep.21897

[b11] Thompson A. J. . Stimulation of the interleukin-1 receptor and Toll-like receptor 2 inhibits hepatitis B virus replication in hepatoma cell lines *in vitro*. Antivir. Ther 14, 797–808 (2009). 19812442 10.3851/IMP1294

[b12] Cardin R. D. . Amphipathic DNA polymers exhibit antiviral activity against systemic murine Cytomegalovirus infection. Virol J 6, 214 (2009). 19954538 10.1186/1743-422X-6-214 PMC2794273

[b13] Davis H. L. . CpG DNA is a potent enhancer of specific immunity in mice immunized with recombinant hepatitis B surface antigen. J Immunol 160, 870–876 (1998). 9551923

[b14] Kariko K., Buckstein M., Ni H. & Weissman D. Suppression of RNA recognition by Toll-like receptors: the impact of nucleoside modification and the evolutionary origin of RNA. Immunity 23, 165–175 (2005). 16111635 10.1016/j.immuni.2005.06.008

[b15] Judge A. D., Bola G., Lee A. C. & MacLachlan I. Design of noninflammatory synthetic siRNA mediating potent gene silencing *in vivo*. Mol Ther 13, 494–505 (2006). 16343994 10.1016/j.ymthe.2005.11.002

[b16] Robbins M. . 2′-O-methyl-modified RNAs act as TLR7 antagonists. Mol Ther 15, 1663–1669 (2007). 17579574 10.1038/sj.mt.6300240

[b17] Zust R. . Ribose 2′-O-methylation provides a molecular signature for the distinction of self and non-self mRNA dependent on the RNA sensor Mda5. Nat Immunol 12, 137–143 (2011). 21217758 10.1038/ni.1979 PMC3182538

[b18] Broering R. . Chemical modifications on siRNAs avoid Toll-like-receptor-mediated activation of the hepatic immune system *in vivo* and *in vitro*. Int. Immunol, 26, 35–46 (2013). 24065781 10.1093/intimm/dxt040

[b19] Devarkar S. C. . Structural basis for m7G recognition and 2′-O-methyl discrimination in capped RNAs by the innate immune receptor RIG-I. Proc Natl Acad Sci USA 113, 596–601 (2016). 26733676 10.1073/pnas.1515152113 PMC4725518

[b20] Matsumura T. . Amphipathic DNA polymers inhibit hepatitis C virus infection by blocking viral entry. Gastroenterology 137, 673–681 (2009). 19394333 10.1053/j.gastro.2009.04.048 PMC2803092

[b21] Kondo Y., Ninomiya M., Kakazu E., Kimura O. & Shimosegawa T. Hepatitis B surface antigen could contribute to the immunopathogenesis of hepatitis B virus infection. ISRN Gastroenterol 2013, 935295 (2013). 10.1155/2013/935295 PMC3562682 23401786

[b22] Geary R. S., Norris D., Yu R. & Bennett C. F. Pharmacokinetics, biodistribution and cell uptake of antisense oligonucleotides. Adv Drug Deliv Rev 87, 46–51 (2015). 25666165 10.1016/j.addr.2015.01.008

[b23] Geary R. S. . Pharmacokinetics of a tumor necrosis factor-alpha phosphorothioate 2′-O-(2-methoxyethyl) modified antisense oligonucleotide: comparison across species. Drug Metab Dispos 31, 1419–1428 (2003). 14570775 10.1124/dmd.31.11.1419

[b24] Geary R. S., Leeds J. M., Henry S. P., Monteith D. K. & Levin A. A. Antisense oligonucleotide inhibitors for the treatment of cancer: 1. Pharmacokinetic properties of phosphorothioate oligodeoxynucleotides. Anticancer Drug Des 12, 383–393 (1997). 9236854

[b25] Brikh C. . Therapeutic effect against hepatitis B of various nucleic acid polymers in the chronic DHBV infection model. Journal of Hepatology 62, S518–S518 (2015).

[b26] Bennett C. F. & Swayze E. E. RNA targeting therapeutics: molecular mechanisms of antisense oligonucleotides as a therapeutic platform. Annu Rev Pharmacol Toxicol 50, 259–293 (2010). 20055705 10.1146/annurev.pharmtox.010909.105654

[b27] Yu R. Z., Grundy J. S. & Geary R. S. Clinical pharmacokinetics of second generation antisense oligonucleotides. Expert Opin Drug Metab Toxicol 9, 169–182 (2013). 23231725 10.1517/17425255.2013.737320

[b28] Bazinet M. . Update on the safety and efficacy of REP 2139 monotherapyand subsequent combination therapy with pegylated interferon alpha-2a in caucasian patients with chronic HBV/HDV co-infection Journal of Hepatology 64, S584 (2016).

[b29] Bazinet M. . Preliminary safety and efficacy of REP 2139-Mg or REP 2165-Mg used in combination with tenofovir disoproxil fumarate and pegylated interferon alpha 2a in treatment naïve Caucasian patients with chronic HBeAg negative HBV infection. Hepatology 64, 1122A (2016).

[b30] Krug A. . Identification of CpG oligonucleotide sequences with high induction of IFN-alpha/beta in plasmacytoid dendritic cells. Eur J Immunol 31, 2154–2163 (2001). 11449369 10.1002/1521-4141(200107)31:7<2154::aid-immu2154>3.0.co;2-u

[b31] Knolle P. A. & Gerken G. Local control of the immune response in the liver. Immunol. Rev 174, 21–34 (2000). 10807504 10.1034/j.1600-0528.2002.017408.x

[b32] Pichlmair A. . RIG-I-mediated antiviral responses to single-stranded RNA bearing 5′-phosphates. Science 314, 997–1001 (2006). 17038589 10.1126/science.1132998

[b33] Gitlin L. . Essential role of mda-5 in type I IFN responses to polyriboinosinic:polyribocytidylic acid and encephalomyocarditis picornavirus. Proc Natl Acad Sci USA 103, 8459–8464 (2006). 16714379 10.1073/pnas.0603082103 PMC1464000

[b34] Broering R. . Long-term stimulation of Toll-like receptor 3 in primary human hepatocytes leads to sensitization for antiviral responses induced by poly I:C treatment. J Viral Hepat 21, 480–490 (2014). 24750363 10.1111/jvh.12174

[b35] Lutterbeck M. . Toll-like receptor 3 activation of human non-parenchymal liver cells induces an antiviral state against HCV which is mediated by type I and type III interferons. Hepatology 58, 1183A–1183A (2013).

[b36] Thomas E. . HCV infection induces a unique hepatic innate immune response associated with robust production of type III interferons. Gastroenterology 142, 978–988 (2012). 22248663 10.1053/j.gastro.2011.12.055 PMC3435150

[b37] Yoneda M. . Hepatitis B Virus and DNA Stimulation Trigger a Rapid Innate Immune Response through NF-kappaB. J Immunol 197, 630–643 (2016). 27288535 10.4049/jimmunol.1502677

[b38] Stelma F. . Cytokine responses in chronic hepatitis B patients dosed with the nucleic-acid polymer REP2139-Ca. Journal of Hepatology 62, S567–S567 (2015).

[b39] Jiang M. . Toll-like receptor-mediated immune responses are attenuated in the presence of high levels of hepatitis B virus surface antigen. J Viral Hepat, 21, 860–872 (2014). 24498958 10.1111/jvh.12216

[b40] Werner M. . All-In-One: Advanced preparation of Human Parenchymal and Non-Parenchymal Liver Cells. PLoS. One 10, e0138655 (2015). 26407160 10.1371/journal.pone.0138655 PMC4583235

[b41] Bustin S. A. . The MIQE guidelines: minimum information for publication of quantitative real-time PCR experiments. Clin. Chem 55, 611–622 (2009). 19246619 10.1373/clinchem.2008.112797

