# Supplementary material for: Nucleic acid-based polymers effective against hepatitis B Virus infection in patients don’t harbor immunostimulatory properties in primary isolated liver cells
Source: Sci Rep. 2017 Mar 8;7:43838. doi: 10.1038/srep43838 (PMC5341074; doi:10.1038/srep43838)
Supplement: Supplemental Figures [file srep43838-s1.pdf]

Nucleic acid-based polymers effective against hepatitis B Virus infection in patients don't harbor immunostimulatory properties in primary isolated liver cells

Catherine Isabell Real[1,2], Melanie Werner[1], Andreas Paul[2], Guido Gerken[1], Joerg Friedrich Schlaak[1,3], Andrew Vaillant[4]*, Ruth Broering[1]*

[1] Department of Gastroenterology and Hepatology, University Hospital at the University Duisburg-Essen, Essen, Germany

[2] Department of General-, Visceral- and Transplantation Surgery, University Hospital at the University Duisburg-Essen, Essen, Germany

[3] Evangelisches Klinikum Niederrhein GmbH, Duisburg, Germany

[4] Replicor Inc., Montreal, Quebec, Canada

* Co-corresponding authors

SUPPLEMENTAL FIGURES

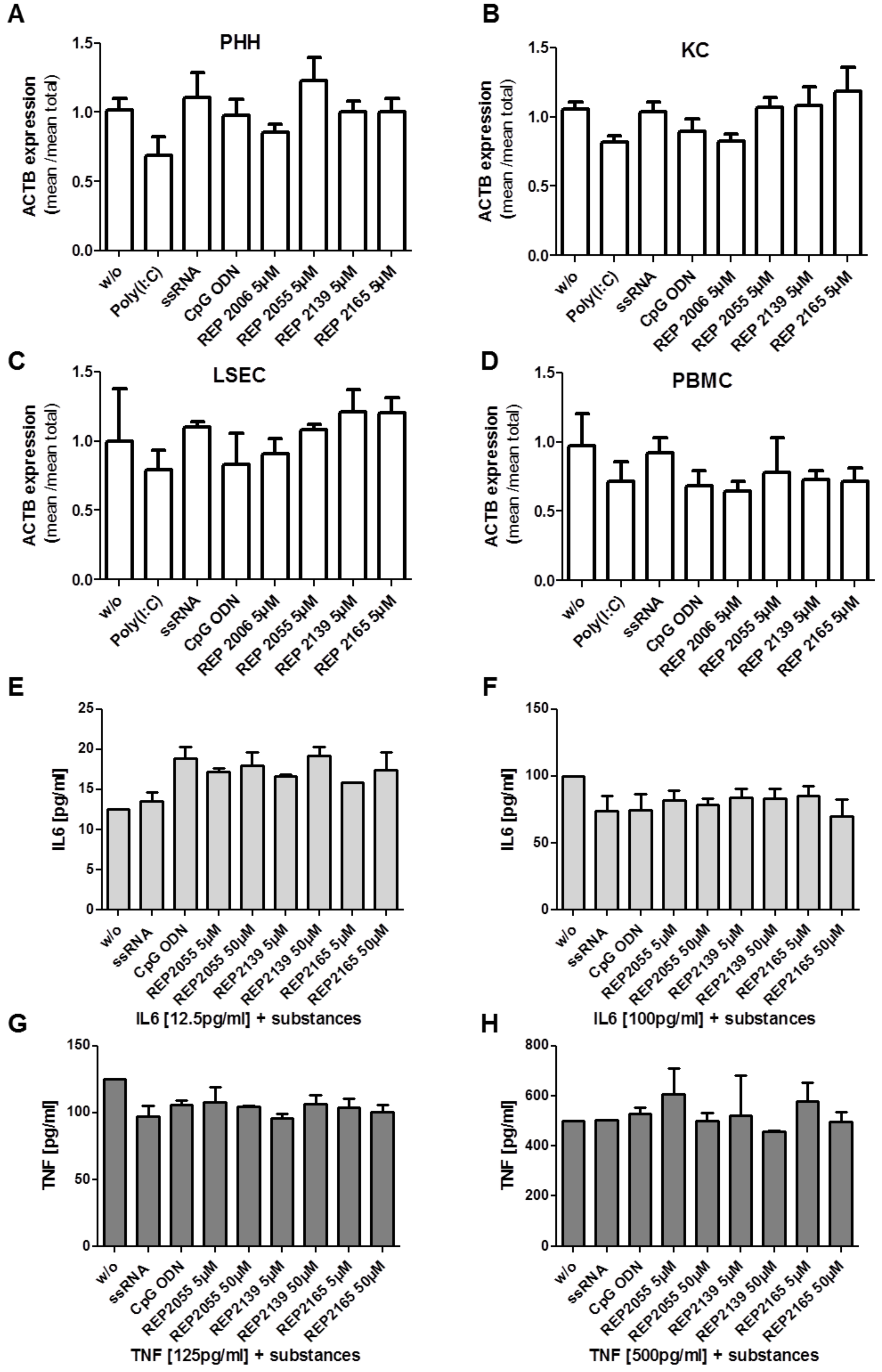

Supplemental figure 1. NAPs do not affect qRT-PCR and ELISA sensitivity. Primary human hepatocytes (PHH, n=3-5) (A), Kupffer cells (KC, n=3-5) (B), liver sinusoidal endothelial cells (LSEC, n=3) (C) and peripheral blood mononuclear cells (PBMC, n=3-5) (D) were stimulated with DNA-based (REP 2006 and REP 2055) and RNA-based (REP 2139 and REP 2165) NAPs or immunostimulatory controls (TLR3 agonist Poly(I:C); TLR7/8 agonist ssRNA40 [ssRNA] and TLR9 agonist CpG ODN2216 [CpG ODN]) for 6h. RNA was extracted, and gene expression of beta actin (ACTB) was analyzed by quantitative reverse transcription polymerase chain reaction (qRT-PCR). To indicate lack of cell toxicity and constant PCR performance, the mean actin expression after treatment was normalized by the total mean of actin expression. The interleukin 6 (IL6) (E, F) and tumor necrosis factor (TNF) (G, H) ELISA was performed according to the manufacturer′s instructions using low (E, G) and high (F, H) concentrations of these cytokines. The standard cytokine was diluted (as indicated) and additionally mixed with different concentrations of NAPs [5µM and 50µM] and TLR ligands (ssRNA and ODN [ODN2216]). Values represented mean ± SEM compared to untreated control.

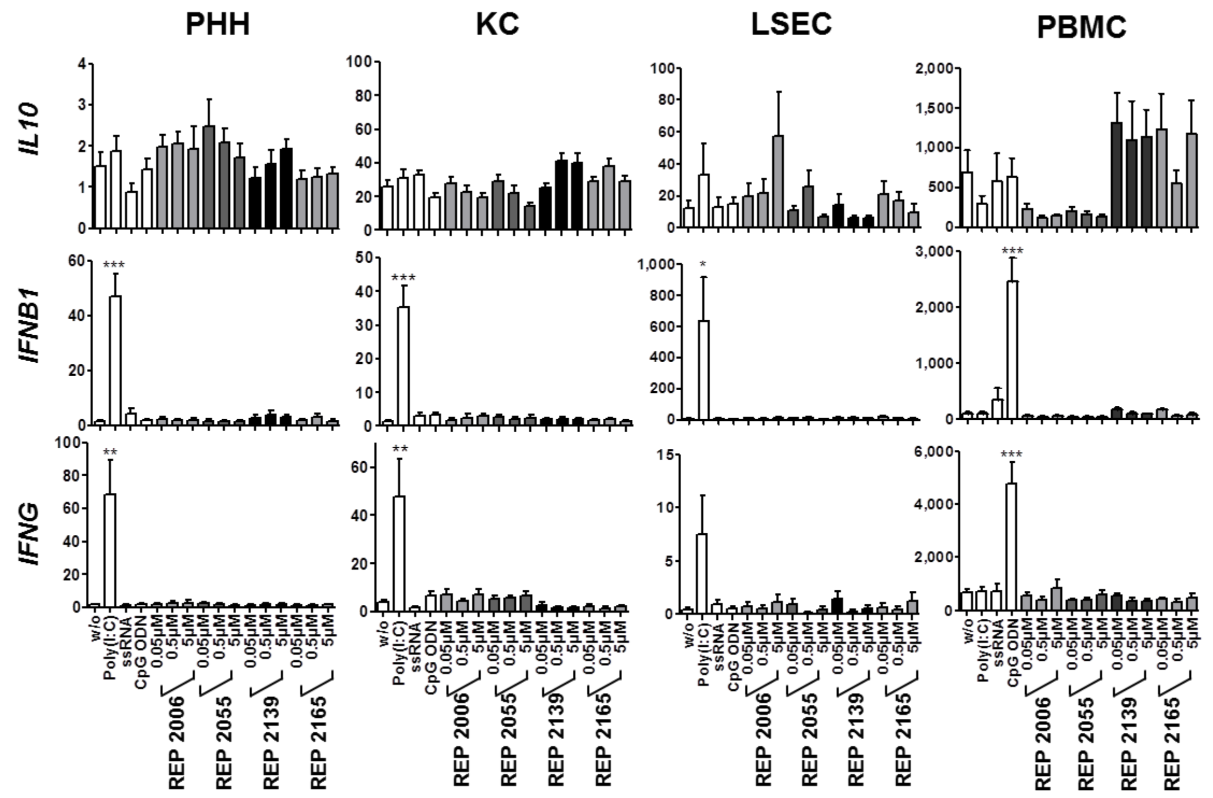

Supplemental figure 2. Cell type-specific expression of innate immune genes in response to NAP treatment *in vitro*. Primary human hepatocytes (PHH, n=3-5), Kupffer cells (KC, n=3-5), liver sinusoidal endothelial cells (LSEC, n=3-6) and peripheral blood mononuclear cells (PBMC, n=3-6) were stimulated with DNA-based (REP 2006 and REP 2055) and RNA-based (REP 2139 and REP 2165) NAPs or immunostimulatory controls (TLR3 agonist Poly(I:C); TLR7/8 agonist ssRNA40 [ssRNA] and TLR9 agonist CpG ODN2216 [CpG ODN]) for 6h. RNA was extracted, and gene expression of interleukin 10 (IL10), interferon beta 1 (IFNB1) and interferon gamma (IFNG) was assessed by quantitative reverse transcription polymerase chain reaction (qRT-PCR). Values represented mean ± SEM (normalized to 100,000 copies of beta actin (ACTB) mRNA). Statistically significant changes compared to untreated controls are reported for $p<0.05$ (*), $p<0.01$ (**), $p<0.001$ (***); w/o, without treatment.

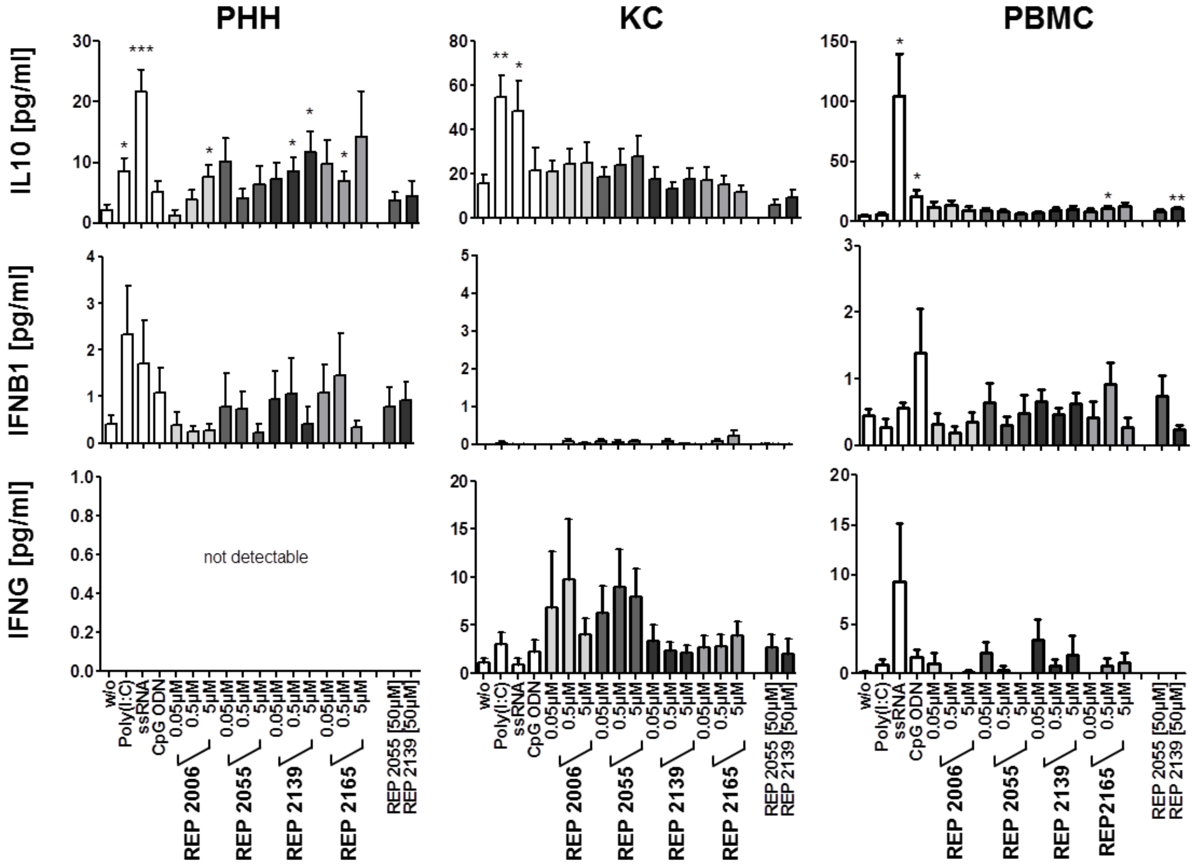

Supplemental figure 3. Cell type-specific cytokine secretion in response to NAP treatment *in vitro*. Primary human hepatocytes (PHH, n=3-5), Kupffer cells (KC, n=3-6) and peripheral blood mononuclear cells (PBMC, n=3) were stimulated with DNA-based (REP 2006 and REP 2055) and RNA-based (REP 2139 and REP 2165) NAPs or immunostimulatory controls (TLR3 agonist Poly(I:C); TLR7/8 agonist ssRNA40 [ssRNA] and TLR9 agonist CpG ODN2216 [CpG ODN]) for 24h. Supernatants were collected and secretion of interleukin 10 (IL10), interferon beta 1 (IFNB1) and interferon gamma (IFNG) was quantified by enzyme-linked immunosorbent assay (ELISA). Values represented mean ± SEM. Statistically significant changes compared to untreated controls are reported for p<0.05 (*), p<0.01 (**), p<0.001 (***); w/o, without treatment.
